# Supplementary material for: Assessment of Some Risk Factors and Biological Predictors in the Post COVID-19 Syndrome in Asthmatic Patients
Source: J Pers Med. 2023 Dec 22;14(1):21. doi: 10.3390/jpm14010021 (PMC10820086; doi:10.3390/jpm14010021)
Supplement: Supplementary file 1 [file jpm-14-00021-s001.zip › jpm-2751664-supplementary.pdf]

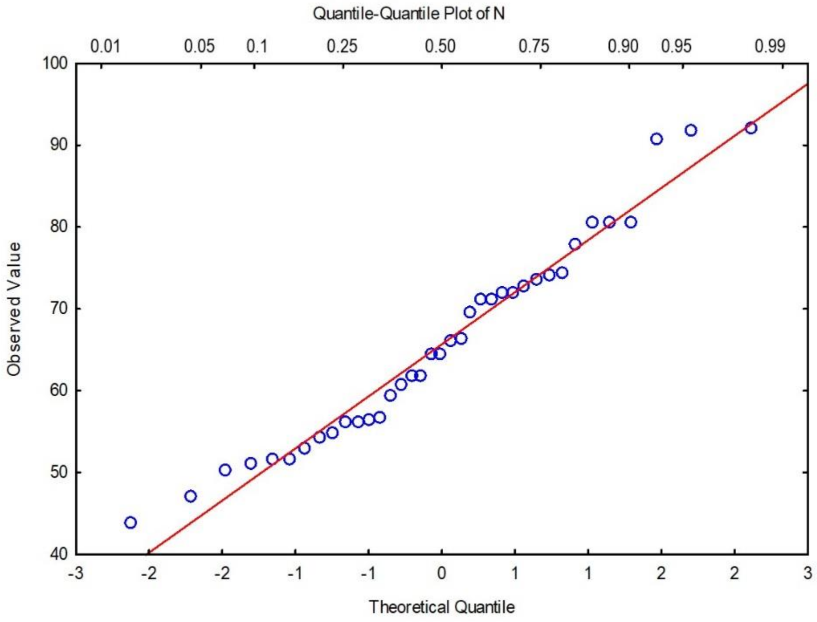
Quantile-Quantile Plot of N
0.01 0.05 0.1 0.25 0.50 0.75 0.90 0.95 0.99
100
90
80
70
60
50
40
Observed Value
-3 -2 -2 -1 -1 0 1 1 2 2 3
Theoretical Quantile

(a)

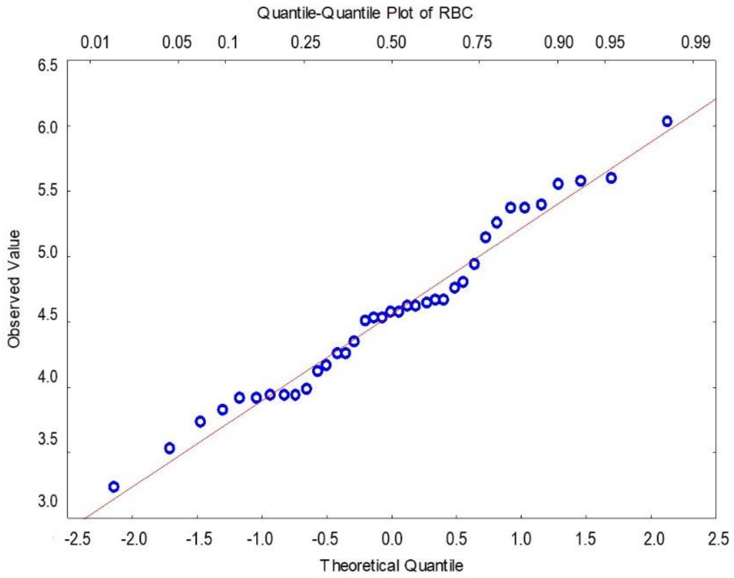
Quantile-Quantile Plot of RBC
0.01 0.05 0.1 0.25 0.50 0.75 0.90 0.95 0.99
6.5
6.0
5.5
5.0
4.5
4.0
3.5
3.0
Observed Value
-2.5 -2.0 -1.5 -1.0 -0.5 0.0 0.5 1.0 1.5 2.0 2.5
Theoretical Quantile

(b)

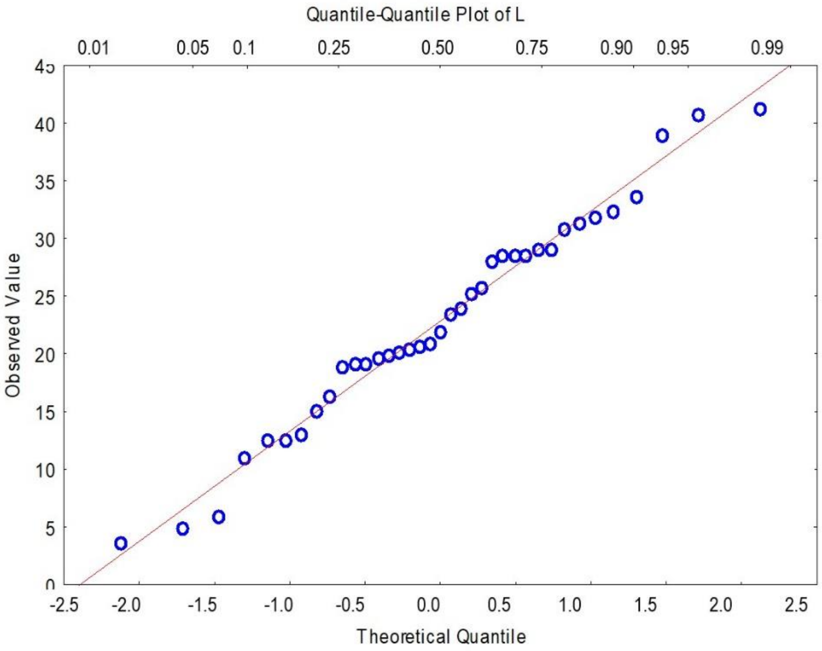

(c)

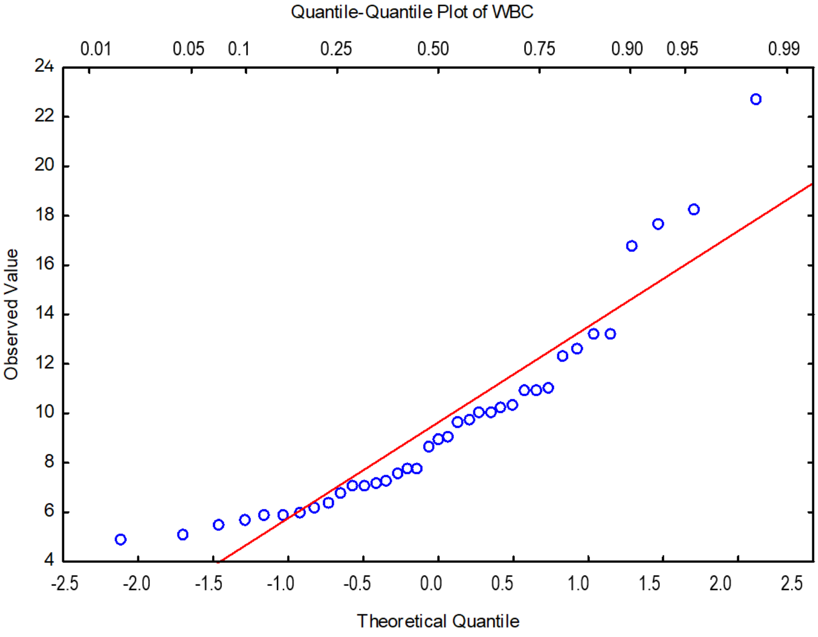

(d)

Figure S1. (a–d). Q-Q plot for the distribution of the values of hematological parameters (N, RBC, L, WBC) in patients with asthma and PCI.

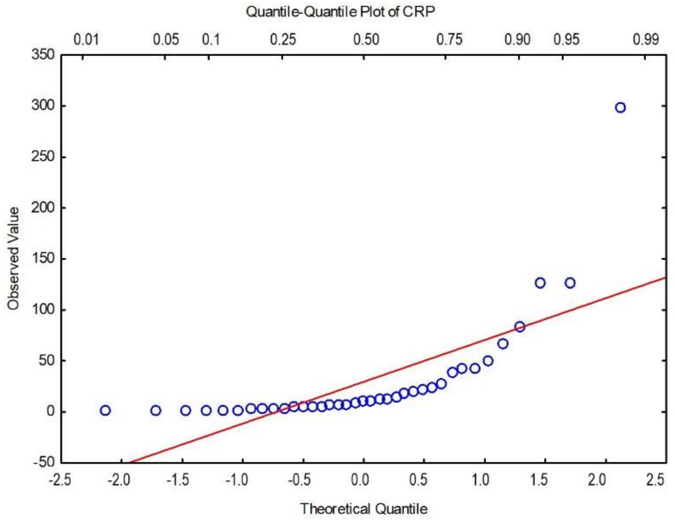
Quantile-Quantile Plot of CRP
0.01 0.05 0.1 0.25 0.50 0.75 0.90 0.95 0.99
350
300
250
200
150
100
50
0
-50
Observed Value
-2.5 -2.0 -1.5 -1.0 -0.5 0.0 0.5 1.0 1.5 2.0 2.5
Theoretical Quantile

(a)

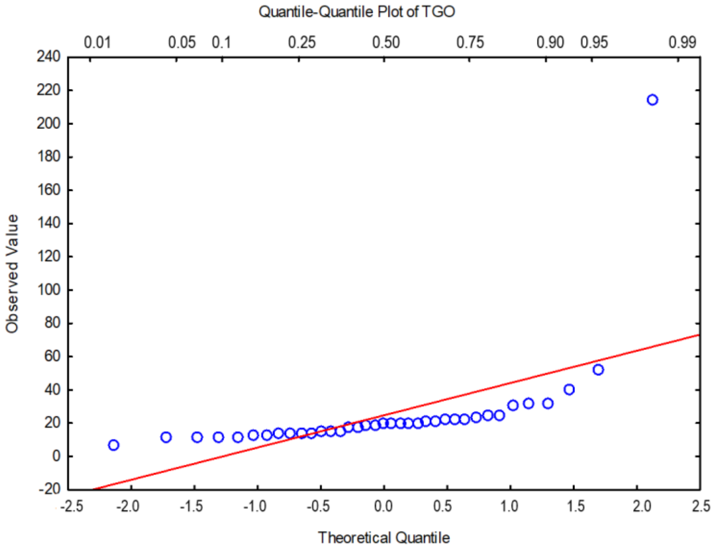
Quantile-Quantile Plot of TGO
0.01 0.05 0.1 0.25 0.50 0.75 0.90 0.95 0.99
240
220
200
180
160
140
120
100
80
60
40
20
0
-20
Observed Value
-2.5 -2.0 -1.5 -1.0 -0.5 0.0 0.5 1.0 1.5 2.0 2.5
Theoretical Quantile

(b)

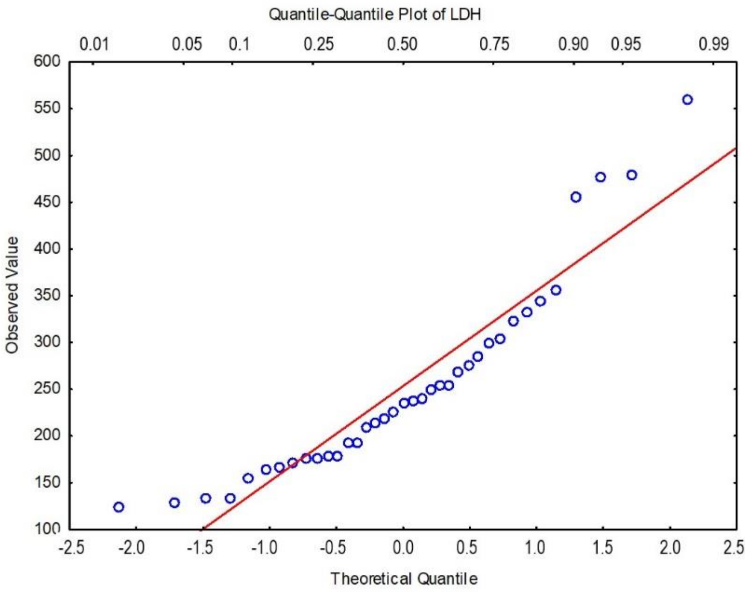
Quantile-Quantile Plot of LDH
0.01 0.05 0.1 0.25 0.50 0.75 0.90 0.95 0.99
600 550 500 450 400 350 300 250 200 150 100
Observed Value
-2.5 -2.0 -1.5 -1.0 -0.5 0.0 0.5 1.0 1.5 2.0 2.5
Theoretical Quantile

(c)

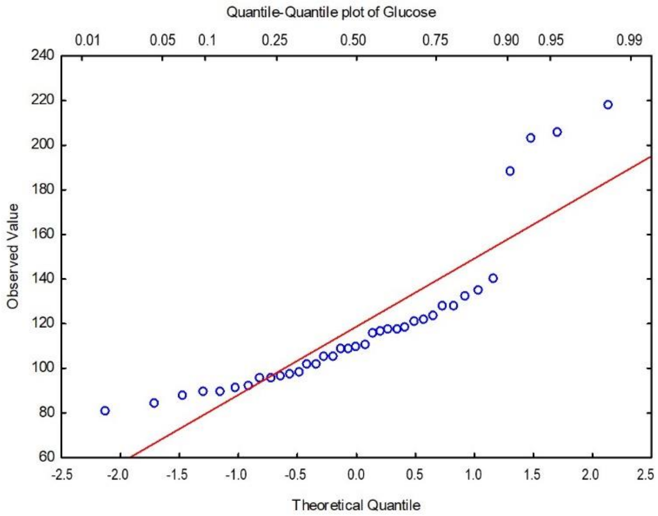
Quantile-Quantile plot of Glucose
0.01 0.05 0.1 0.25 0.50 0.75 0.90 0.95 0.99
240 220 200 180 160 140 120 100 80 60
Observed Value
-2.5 -2.0 -1.5 -1.0 -0.5 0.0 0.5 1.0 1.5 2.0 2.5
Theoretical Quantile

(d)

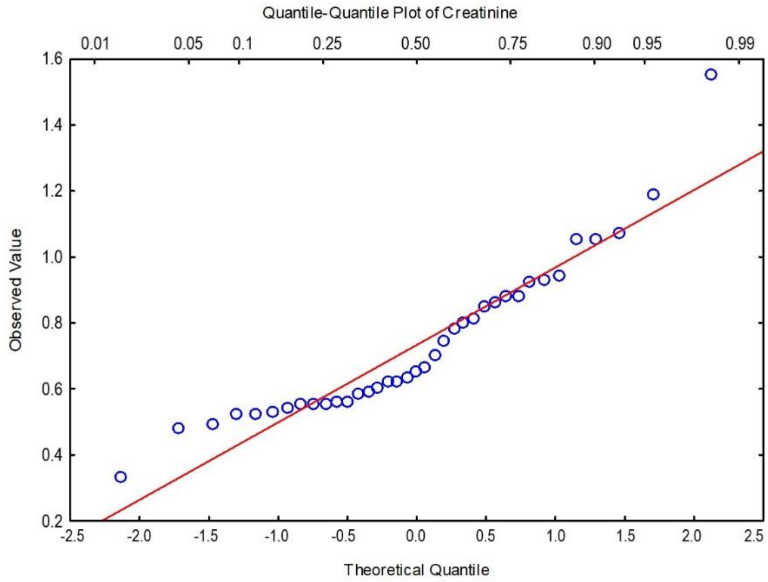

(e)

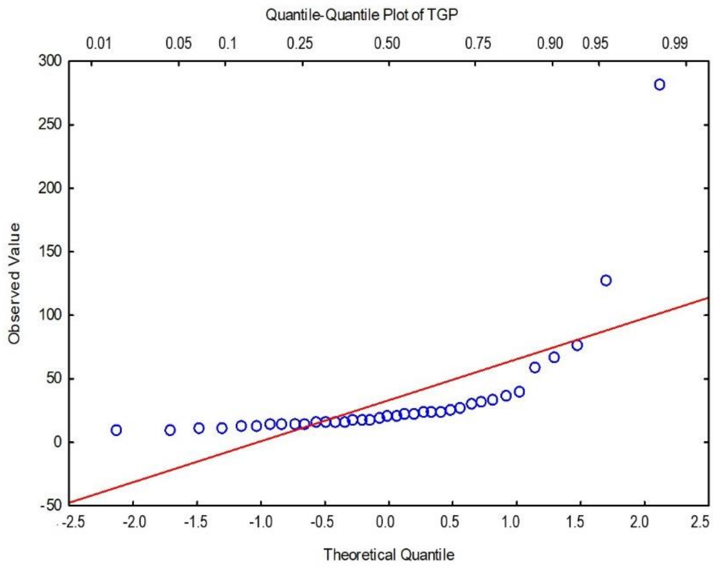

(f)

Figure S2. (a–f). Q-Q plot for the distribution of the values of the biochemical and hematological parameters in patients with asthma and PCI
